# Age trends in the prevalence of cervical squamous intraepithelial lesions among HIV-positive women in Cameroon: a cross-sectional study

**DOI:** 10.1186/1756-0500-5-590

**Published:** 2012-10-29

**Authors:** Julius Atashili, William C Miller, Jennifer S Smith, Peter M Ndumbe, George M Ikomey, Joseph Eron, Allen C Rinas, Evan Myers, Adaora A Adimora

**Affiliations:** 1 University of Buea, Box 63, Molyko Buea, Cameroon; 2 University of Yaounde I, Yaounde, Cameroon; 3 University of North Carolina at Chapel Hill, Chapel Hill, North Carolina, USA

## Abstract

**Background:**

Cervical squamous intra-epithelial lesions (SIL) are more frequent in HIV-positive women overall. However the appropriate age at which to begin and end cervical cancer screening for early detection of lesions in HIV-positive women is not clear. We assessed the age-specific prevalence of any SIL and SIL requiring colposcopy in HIV-positive women in Cameroon.

**Methods:**

We enrolled, interviewed and conducted conventional cervical cytology in 282 women, aged 19–68 years, initiating antiretroviral therapy in three clinics in Cameroon. In bivariable analyses, the crude relationship between age and the presence of lesions was assessed using locally weighted regression (LOWESS) methods. In multivariate analyses, generalized linear models with prevalence as the outcome, an identity link and a binomial distribution, were used to estimate prevalence differences. Bias analyses were conducted to assess the potential effect of inaccuracies in cytology.

**Results:**

SIL were detected in 43.5% of the 276 women with satisfactory samples, 17.8% of whom had ASC-H/HSIL. On average, women aged 26 to 59 tended to have a slightly higher prevalence of any SIL than other women (Prevalence difference PD: 6.5%; 95%CI: -11.4, 24.4%). This PD was a function of CD4 count (heterogeneity test p-value =0.09): amongst patients with CD4 counts less than 200cells/uL, the prevalence was higher in patients aged 26–59, while there was essentially no difference amongst women with CD4 counts greater than 200 cells/uL. ASC-H/HSIL were present in women as young as 19 and as old as 62. Overall the prevalence of ASC-H/HSIL increased by 0.7% (95%CI: -3.8%, 5.1%) per decade increase in age.

**Conclusion:**

Both severe and less severe lesions were prevalent at all ages suggesting little utility of age-targeted screening among HIV-positive women. Nevertheless, the long-term evolution of these lesions needs to be assessed in prospective studies.

## Background

Cervical cancer is the second most common cancer in women worldwide [1]. Although cervical cancer incidence and mortality is higher in HIV-positive women, resource limitations restrict the implementation of systematic screening programs in these women in developing countries. With the recent increase in access to antiretroviral therapy HIV-positive women are expected to live longer, potentially allowing sufficient time for cervical cancer to develop. Targeted screening could potentially alleviate the strain on resources needed to screen these women.

Age has been a common consideration in the targeted screening for precancerous lesions in the general population. Current guidelines for screening the general population of women in the United States (US) suggest screening commence no later than age 21 years, reducing the frequency of screening at age 30 among women with previously negative cytology results and stopping screening at age 65 (or 70 in some guidelines) [2-4]. World Health Organization (WHO) guidelines aimed primarily at resource-limited settings are less stringent, recommending screening begin at age 30, need not be annual and need not be done over the age of 65 [5]. These age considerations may not necessarily be ideal for HIV-positive women among whom higher human papilloma virus (HPV) prevalence, higher HPV persistence, and a faster progression of lesions [6-11] could mean an earlier occurrence and or a longer persistence of precancerous lesions. The optimal age for screening in HIV-positive individuals could thus be younger than for women in the general population.

We describe here the age-specific prevalence of lesions in HIV-positive women initiating antiretroviral therapy in Cameroon, with the aim of estimating the minimum age at which lesions occur, the age with maximum occurrence and the latest age at which lesions occur.

## Methods

### Study design and population

In this cross-sectional study, HIV-positive women were recruited from three HIV-care clinics in Cameroon: the Bamenda Provincial Hospital AIDS Treatment Center (ATC), the Limbe Provincial Hospital ATC and the Nylon District Hospital ATC in Douala. These are all located in urban areas in Cameroon and provide regular care to patients from surrounding urban areas and peripheral rural areas. Consecutive HIV-positive women receiving care in these clinics, between August and September 2008, were invited to participate in the study. Women aged 18 years or more, who initiated HAART within a year of study enrollment and consenting to study procedures were eligible. Women who were either pregnant, bleeding due to menses or had a previous total hysterectomy were excluded. After obtaining written consent from each participant, socio-demographic and clinical data were collected using a structured interview, a clinical examination and a review of medical records. Cervical cell samples were then collected using Ayre’s spatula, and smeared into two pre-labeled slides.

Conventional cytology slides collected were transported to the laboratory of the Center for the Study and Control of Communicable Diseases (CSCCD) in Yaounde, Cameroon where they were stained by the Papanicolau’s method and examined under the microscope by a trained cytologist. The stained slides were observed under the microscope (at 400X) and then scored according to the Bethesda 2001 system, as unsatisfactory; negative; atypical squamous cells of uncertain significance (ASCUS); low-grade squamous intraepithelial lesions (LSIL); atypical squamous cells, cannot exclude high grade lesions (ASC-H); high-grade SIL (HSIL); or invasive cervical cancer [12]. For quality control purposes, both research assistants and cytologist received specific training related to the study, two slides were made and analyzed for each patient (the most severe result was considered the final result, in case of differences between both slides), and slides with lesions were double-checked by a cytologist external to the study (differences were resolved by consensus). Furthermore, a subset of 10% slides were reviewed by an experienced cytologist at the University of North Carolina at Chapel Hill – the percentage agreement was on the presence of lesions was 76%, (kappa=0.49) while the percentage agreement on lesions being ASC-H/HSIL was 60% (kappa=0.26). The potential impact of these limitations with conventional cytology were assessed in sensitivity (bias) analyses [13].

The study was approved by the Cameroon National Ethics Committee and the University of North Carolina at Chapel Hill's Biomedical IRB (USA).

### Data analysis

Data collected were entered into MS Access interface on Epi-info 2000. Statistical analysis were conducted using SAS version 9.2 (SAS institute inc, Cary NC) and Stata version 10 (Stata corps, Texas USA). Two outcomes were considered for this analysis: 1) Prevalent cervical lesions (defined as the presence of any cervical epithelial lesions); 2) Prevalent ASC-H/HSIL. Age (in years) was the independent predictor considered. Other covariates considered in this analysis included marital status, education level, parity, history of hormonal contraception, smoking history, CD4 count, and AIDS clinical stage. Univariable distributions of these characteristics were determined by computing means, median and ranges (for continuous variables) and proportions at different levels (for categorical variables).

In bivariable analyses, the crude relationship between each outcome and age was assessed using locally weighted regression (LOWESS) methods with a smoothing parameter of 0.5 [13]. In subsequent analyses, we used generalized linear models with prevalence as the outcome, an identity link and a binomial distribution, as we sought to estimate prevalence differences [13]. We explored coding age as a continuous variable (linear or quadratic) or coding age as a categorical variable with cut-offs based on the LOWESS-smoothed curve. For each outcome, the coding of age that resulted in the best model fit (or least deviance), as assessed by a likelihood ratio test (for nested models) or the Akaike’s Information criterion (for non-nested models) was selected. Age coded as a binary variable (age 26–59 or not) had the best fit in modeling the association of age and any lesion, while age coded as a continuous linear variable had the best fit in modeling the association of age and ASC-H/HSIL.

In multivariable analysis all covariates other than age and the outcomes were assessed as potential modifiers of the prevalence difference. Each covariate was coded as a binary variable and a product interaction term created between age and each covariate. Covariates were considered modifiers if a likelihood ratio test of the product interaction term had a p-value less than 0.1, (a higher cut-off point set a priori to account for the low power associated with tests of homogeneity) or if the stratum-specific prevalence differences varied by 20% or more.

Although all covariates were also considered as potential confounders, a Directed Acyclic Graph (DAG) analysis revealed that none of the variables should be considered a confounder [13]. We attempted to mathematically estimate the minimum and maximum ages at which cervical lesions are present as well as the age with the maximum prevalence of lesions assuming a quadratic relationship between age and prevalent lesions. Only the age of maximum prevalence could however be estimated as all other models resulted in extrapolations beyond biologically plausible ages. Age was centered in these models to allow for a meaningful interpretation of all model parameters [13].

## Results

Altogether 282 women were enrolled in this study. Participants’ age ranged from 19 to 68 years (with a mean of 36 years). The median CD4 count was 179 cells/microliter (interquartile range: 100 to 271). SIL were detected in 43.5% of the 276 women with satisfactory samples: 0.7% as ASCUS, 25.0% as LSIL, 14.5% as ASC-H, and 3.3% as HSIL.

### Prevalence of lesions by age

**Figure 1:**
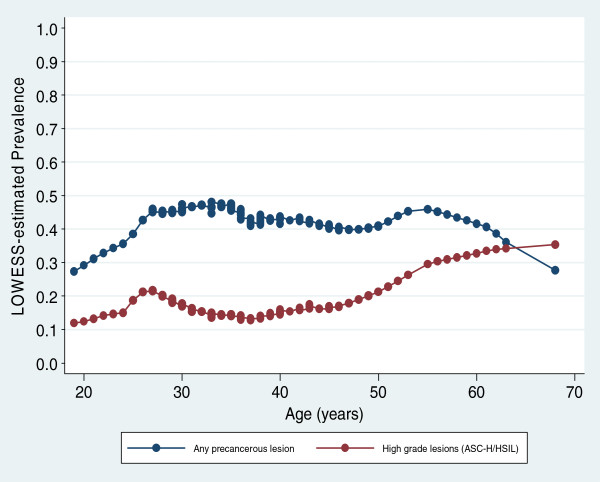
Trends in age-specific prevalence of precancerous lesions and ASC_H/HSIL in 276 women initiating HAART in Cameroon (estimates based on locally weighted regression models).

The age of participants with no lesions ranged from 19 to 68 (with a mean of 36.3) years while that of participants with any lesion ranged from 19 to 62 (with a mean of 35.7) years. The prevalence of any lesion tended to increase from age 19 to a peak at about 25 years (Table 1), from which it stabilized between 40% and 50% until the age of 60, after which it reduced among the small number of women surveyed (Figure 1).

**Table 1:** Age-specific prevalence of cervical precancerous epithelial lesion in 276 women initiating HAART in Cameroon

**Age (Years)**	**N**	**Prevalence of any lesion**		**Prevalence of ASC-H/HSIL**	
		**%**	**95% CI**	**%**	**95% CI**
18-24	16	31.3	11.0, 58.7	6.3	01.6, 30.2
25-34	124	45.2	36.2, 54.3	18.5	12.1, 26.5
35-44	80	43.8	32.7, 55.3	15.0	08.0, 24.7
45-54	42	40.5	25.6, 56.7	16.7	07.0, 31.4
55-59	7	57.1	18.4, 90.1	42.9	09.9, 81.6
60+	6	33.3	04.3, 77.7	33.3	04.3, 77.7

On an average women aged 26 to 59 had a slightly higher prevalence than relatively younger or older women (Prevalence difference PD: 6.5%; 95%CI: -11.4, 24.4%). However this PD was a function of CD4 count (heterogeneity test p-value =0.09). Amongst patients with CD4 counts less than 200cells/uL, women aged 25–59 had a substantially higher prevalence (PD= 21.0%; 95% CI: -0.8%, 42.8%). In contrast, there was only a little difference in prevalence by age among women with CD4 counts greater than 200 cells/uL (PD= −9.8%; 95%CI: -37.8%, 18.3%).

**Figure 2:**
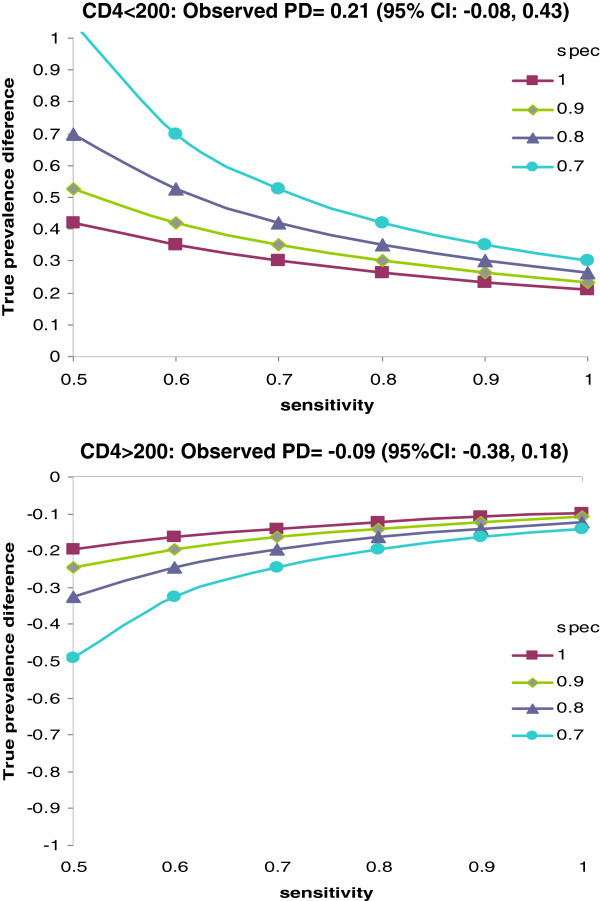
Sensitivity analysis of outcome misclassification on the observed prevalence difference between age groups (26–59 versus 18–25 and 60+ years) by CD4 count.

We conducted bias analyses assessing what the true population prevalence difference of lesions could be considering the inaccuracies in conventional cytology. The misclassification of the outcome resulting from these inaccuracies was assumed to be non-differential as the cytologist was masked from participants’ ages. Our analysis showed that a lower cytology sensitivity or specificity would mean that the study tended to underestimate the magnitude of the prevalence difference between age groups (Figure 2). For example with a sensitivity of 70% and a specificity of 90% among women with CD4 counts less than 200cells/uL, the prevalence of lesions in women aged 25–59 could be 35% higher than in younger or older women. A similar sensitivity and specificity among women with CD4 counts more than 200cells/uL, could correspond to a 16.3% lower prevalence of lesions in women aged 25–59 compared to younger or older women.

Assuming a quadratic relationship between age and the prevalence of SIL, the age with maximum prevalence was estimated to be 34.9 (95% CI: 11.6, 58.1) years.

### Prevalence of ASC-H/HSIL by age

The age of participants with ASC-H/HSIL ranged from 19 to 62 (with a mean of 36.5) years. In contrast to any lesion, the age-specific prevalence of ASC_H/HSIL increased slowly but more or less monotonically with age (Figure 1). On an average, the prevalence of ASC-H/HSIL increased by 0.7% (95%CI: -3.8%, 5.1%) per decade increase in age. The age-specific prevalence of ASC-H/HSIL did not appear to differ by CD4 count.

## Discussion

Data on age-specific prevalence of SIL are needed if age-targeted screening is to be considered in HIV-positive women. In this paper, we show that while the prevalence of SIL appeared highest in the third and fourth decades of life, and the prevalence of ASC-H/HSIL gradually increased with age, the prevalence of lesions did not appear to be age-limited.

The epidemiology of SIL in the general population of (mainly HIV-negative) women has been at the origin of age-targeted screening in these women. Studies conducted in the 1980s, documented the prevalence of lesions in young women [14-16]. Subsequent studies showed that the peak of occurrence of precancerous lesions was in the third to fourth decade while malignant lesions tended to occur later in the fourth or fifth decade [17-20]. Lesions also appeared to be less frequent in women far past menopause [21].

In this study limited to HIV-positive women, the prevalence of lesions was only slightly higher in all women aged 25–59 compared to other women. This suggests that, unlike in HIV-negative women, age only may not be a good criterion for targeted screening. Age differences in the prevalence of lesions, however, appeared to depend on CD4 counts. Amongst women with low CD4 counts, middle-aged women had a higher prevalence than younger or older women, suggesting that screening efforts are particularly needed in these women. To the best of our knowledge few studies have discussed the age-specific prevalence of lesions and severe lesions in HIV-positive women. Unlike our study in which the prevalence of ASC-H/HSIL lesions increased with age, Parham et al. [22] described an inverse-U trend among 691 HIV-positive women aged 23–49 years in Zambia, with a peak prevalence of HSIL/invasive cancer between age 35 and 40 years. It is not clear why these findings differ but the variations in study population age, the relatively low CD4 counts (median of 165) and slightly different outcomes may have contributed to this difference.

While we document prevalent ASC-H/HSIL at all ages in HIV-positive women, it is not clear what the long-term outcome of these lesions would be and this may depend on age. It is conceivable that if lesions were less likely to progress in younger versus older women then targeting older women would be justified or vice versa. Prospective studies in HIV-negative women have had inconsistent results: while lesions were more likely to progress in older women in some studies [23] the majority of studies noted similar progression rates irrespective of age [24-28]. Similar studies need to be conducted in HIV-positive women with limited access to systematic screening.

Our findings are susceptible to bias from misclassification of outcomes as conventional cytology typically has a low sensitivity [29]. Nonetheless, because the cytologists were masked from participants’ age information, these errors are expected to be independent of age (non-differential) potentially biasing our effect estimates towards the null (resulting in an underestimate of the difference in prevalence by age groups).

Secondly, because of the cross-sectional design of the study, the age-specific prevalence described here reflects the age of lesion detection and not necessarily the age of incidence or the age-specific prevalence in the population. Age differences in access to clinics may result in artificially increased prevalence in older women who are more likely to be in the health care system. The latter detection bias is however expected to be minimal in a study population of HIV-positive women in whom access to care is largely driven by worsening HIV disease rather than age. Four in five women in this study had advanced HIV diseases (WHO stage III or IV) and the small number of women aged 50 or more than limited the influence of these women on study estimates.

## Conclusions

In conclusion, cervical precancerous lesions were prevalent at all ages in this population of HIV-positive women, suggesting little utility of age-targeted screening. A better understanding of the value of age-targeted screening would require an assessment of the age-specific long-term evolution of untreated non-severe lesions and treated severe lesions using prospective studies. The potential costs and benefits associated with age-targeted screening will also need to be evaluated in formal cost-effectiveness analyses.

## Abbreviations

ASC-H: Atypical Squamous cells, cannot exclude high grade lesions; ASCUS: Atypical Squamous Cells of Uncertain Significance; ATC: AIDS Treatment Center; CI: Confidence Interval; HAART: Highly Active Antiretroviral Therapy; HIV: Human Immunodeficiency Virus; HPV: Human Papilloma Virus; HSIL: High-grade Squamous Intra-epithelial Lesions; LOWESS: Locally weighted regression; LSIL: Low-grade Squamous Intra-epithelial Lesions; PD: Prevalence difference; SIL: Squamous Intra-epithelial Lesions; WHO: World Health Organisation.

## Competing interests

JA has received honoraria from GSK and research supplies fr*om Digene. J*SS has received research grants, honoraria, or consulting fees during the last three years from GSK, Digene and GenProbe. EM has received research funding and done consulting for Merck & Co.

## Authors' contributions

Study conception and design: JA, JSS, AAA, JE, WCM, EM. Study implementation: JA, JSS, PMN, GMI, ACR. Analysis and review: JA, GMI, ACR, JSS, WCM. Manuscript writing and revisions: JA, PMN, GMI, JSS, AAA, JE, WCM, EM. All authors read and approved the final manuscript.
